# Neurofibromatosis Type 1 Associated with Hashimoto's Thyroiditis: Coincidence or Possible Link

**DOI:** 10.1155/2013/910656

**Published:** 2013-04-18

**Authors:** Junaid Nabi

**Affiliations:** Department of Surgery, Shaheed Suhrawardy Medical College and Hospital, Dhaka 1207, Bangladesh

## Abstract

*Introduction*. Hashimoto's thyroiditis is a common form of chronic autoimmune thyroid disease (AITD) and often coexists with other autoimmune diseases, but Hashimoto's thyroiditis associated with an autosomal dominant neurofibromatosis type 1 is exceedingly rare. *Case Presentation*. A 30-year-old Bengali woman presented to the OPD with complaints of aching pain and tingling sensation in her hands and feet. Physical examination revealed dysmorphic facies, nodular swelling in the neck, cafe-au-lait spots, and neurofibromas covering the entire surface of her body. Her thyroid hormones were within normal limits. Thyroid ultrasound revealed a cystic area in the left lobe of the gland, and ultrasound-guided fine needle aspiration cytology revealed lymphocytic infiltration of the gland, suggesting Hashimoto's thyroiditis. High levels of autoimmune antibodies such as antithyroglobulin and antimicrosomal antibodies confirmed the diagnosis. *Conclusion*. When encountered with a patient of Neurofibromatosis type 1, a physician should be careful about the possibility of a concomitant autoimmune disease. Clinical presentation of neurofibromatosis and Noonan syndrome often overlaps and recent studies have implicated a mutation in NF1 gene in the etiology of NFNS. More extensive reports and further investigations of such patients having combination of neurofibromatosis type 1 and autoimmune thyroiditis will certainly provide better understanding of this link in the near future.

## 1. Introduction

Hashimoto's thyroiditis or goitrous autoimmune thyroiditis is a common form of chronic autoimmune thyroid disease (AITD). The disorder affects up to 2% of the general population [1] and is more common in older women and ten times more frequent in women than in men [2]. Neurofibromatosis type 1 (NF1) is an autosomal dominant neurocutaneous disorder, characterized by neurofibromas, café-au-lait spots, axillary and inguinal freckling, and Lisch nodules in the eye and occasionally associated with optic glioma and difficulties in learning. NF1 is caused by mutation of the NF1 gene on chromosome 17q11.2 [3]. The NF1 gene encodes for neurofibromin, which acts as a tumor suppressor protein. Noonan syndrome (NS) is an autosomal dominant disorder, which was reported first by Kobylinski in 1883 and later described by Noonan and Ehmke in 1963 [4] with incidence estimated at 1 in 1000 to 1 in 2500 live births [5]. It is characterized by unusual triangular-shaped face, micrognathia, hypertelorism, down-slanting eyes, ptosis, low-set ears, webbed neck, congenital heart disease, short stature, chest deformities, and mental retardation [6]. The diagnosis of Noonan syndrome is mainly clinical, with some recent studies showing mutation in the PTPN11 gene to be present in about 50–60% of individuals with Noonan syndrome [7]. NF1 associated with autoimmune diseases is rare. A review of the literature reveals that Hashimoto's thyroiditis associated with NF1 is extremely rare, and only two cases have been reported so far [8, 9]. We present a case of Hashimoto's thyroiditis incidentally detected in a patient with neurofibromatosis type 1 and Noonan phenotype.

## 2. Case Presentation

**Figure 1:**
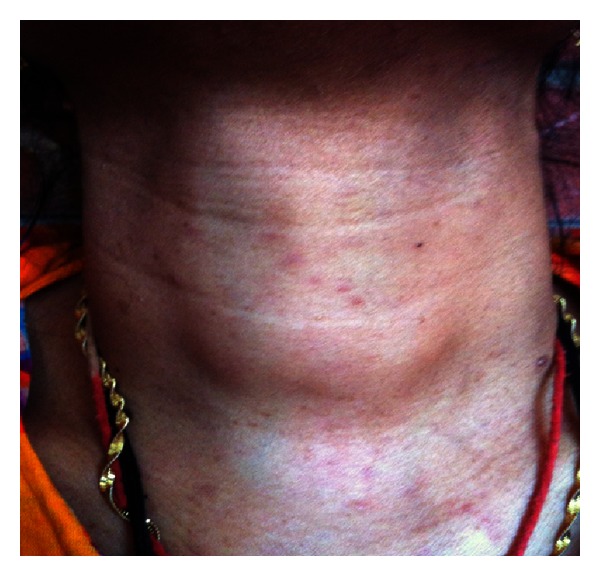
Physical examination revealed a short webbed neck, with a small nodular growth in the left side of the neck which moved with deglutition and was firm in consistency.

**Figure 2:**
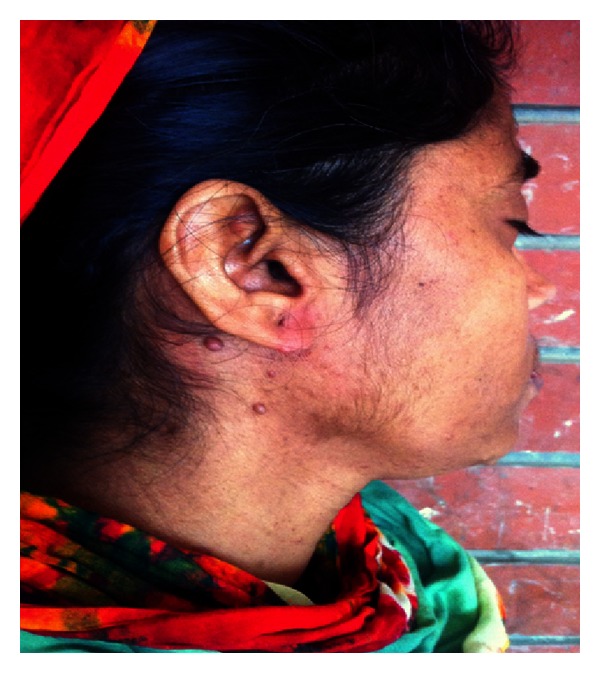
Side view showing dysmorphic facies of Noonan syndrome: low-set ears, micrognathia, and periauricular skin tags.

**Figure 3:**
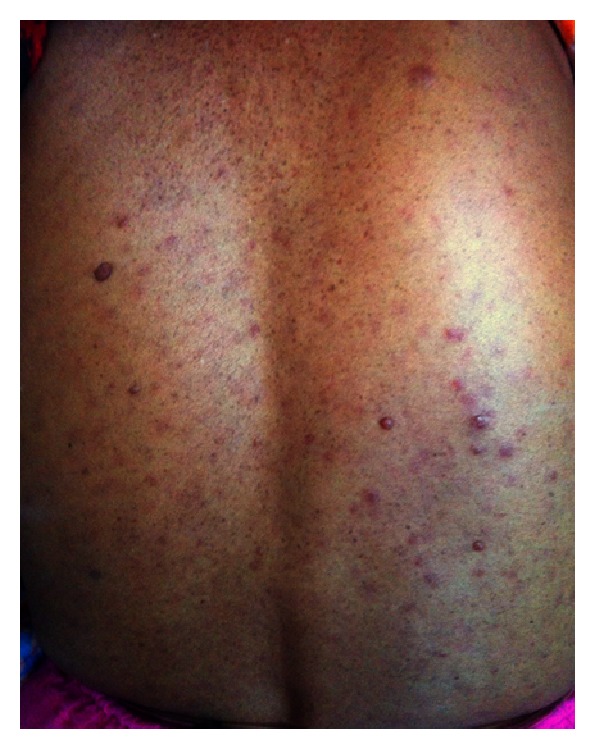
Neurofibromas were spread on the entire surface of the body.

**Figure 4:**
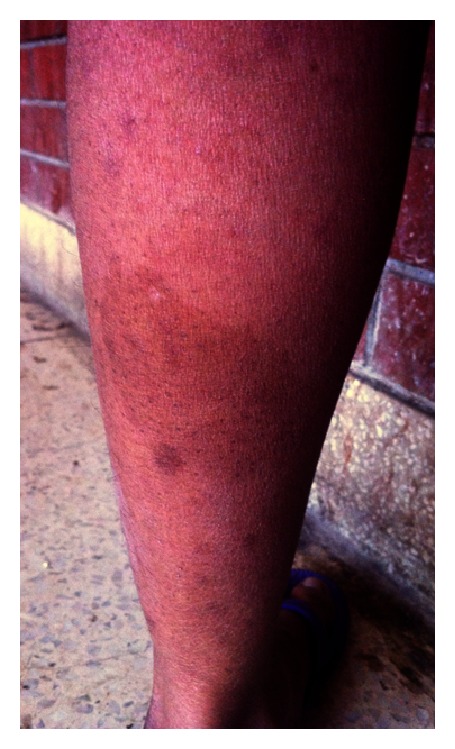
Multiple café-au-lait macules on the calf, the largest one measuring 15 × 8 cms.

A 30-year-old Bengali woman presented at our out-patient department (OPD) because of aching pain and tingling sensation in her hands and feet and recent increase in the number and size of lesions on her skin which were present since birth. She also complained of feeling lethargic with occasional breathlessness and unable to tolerate cold places. Her family history revealed consanguineous marriage of her parents, and her father also had similar skin lesions all over the body. Physical examination revealed a short webbed neck with a small nodular growth in the left side of the neck which moved with deglutition and was firm in consistency (Figure 1) and mild scoliot'ic change in the vertebrae. The patient was 148 cms tall; her face was triangular with a small chin (micrognathia) and a more pronounced forehead; ptotic eyes and ears were set low with periauricular skin tags (Figure 2); neurofibromas cover the entire surface of her body (Figure 3) and café-au-lait macules with the largest one measuring 15 × 8 cms on her left calf (Figure 4) and axillary skin fold freckling. 

The results of a complete blood count, serum biochemistry, and urine analysis were normal. Laboratory examination for karyotype analyses revealed normal female with 46 XX. Our patient was euthyroid at presentation and had serum levels of free thyroxine (T4) at 11.0 *μ*g/dL (normal range was from 5.0 to 13.0 *μ*g/dL), triiodothyronine (T3) at 2.4 pg/mL (normal range was from 1.4 to 4.2 pg/mL), and thyroid stimulating hormone (TSH) levels of 4.77 *μ*IU/mL (normal range was from 0.4 to 5.5 *μ*IU/mL). Thyroid ultrasound (US) revealed a cystic area measuring approximately 2.4 × 2 cm in the left lobe of the gland, not associated with lymphadenopathy. An ultrasound-guided fine needle aspiration cytology (FNAC) was carried out which reported thyroid follicular cells in groups and sheets mixed with lymphocytes, histiocytes, and plasma cells with some of the follicular cells showing Hurtle cell change; the findings suggested the diagnosis of Hashimoto's thyroiditis. Autoimmune antibodies such as antithyroglobulin were at 688 IU/mL (normal range < 100 IU/mL), and anti-microsomal antibodies were >1000 IU/mL (normal range was <35 IU/mL), confirming the diagnosis. US abdomen and electrocardiographic findings were normal, and the echocardiograph reported slight decrease in ejection fraction. 

## 3. Discussion

Hashimoto's thyroiditis has often been shown to coexist with other autoimmune diseases such as type 1 diabetes, celiac disease, rheumatoid arthritis, multiple sclerosis, and vitiligo as well as expressed as part of an autoimmune polyendocrine syndrome type 2 (APS-2), which is usually defined by the occurrence of two or more of following: Addison's disease (always present), AITD, and/or type 1 diabetes [10], in the same patient. However, association of Hashimoto's thyroiditis with neurofibromatosis 1 is exceedingly rare, especially since each of them follows a different pathophysiology. A comprehensive search of published literature revealed two more instances where NF1 was associated with autoimmune diseases, especially with vitiligo and autoimmune thyroiditis [8, 9]. NF1, also known as von Recklinghausen disease, is an autosomal dominant multisystem disorder, which affects approximately 1 in 3500 people [11]. In 1987, seven cardinal diagnostic criteria for NF1 were established [12]. If any two of the following seven criteria are met, a diagnosis of NF1 is made: (a) two or more neurofibromas on or under the skin or one plexiform neurofibroma, (b) freckling of the groin or the axilla (arm pit), (c) six or more café-au-lait spots measuring 5 mm in the greatest diameter in prepubescent individuals and over 15 mm in the greatest diameter in post-pubescent individuals, (d) skeletal abnormalities such as sphenoid dysplasia or thinning of the cortex of the long bones of the body, (e) two or more Lisch nodules (hamartomas of the iris), (f) optic glioma, or (g) a first-degree relative with NF1. These diagnostic criteria are highly specific to adults with NF1. Our patient presented with neurofibromas all over the surface of her body, multiple large café-au-lait spots, and axillary freckling along with a positive family history. The pathophysiology involves a mutation in NF1 gene, which encodes for a tumor suppressor protein neurofibromin, was discovered in 1990, and is located on chromosome 17q11.2 [3]. Abnormal production of neurofibromin suppresses expression of fas-ligand, preventing apoptosis of CD4+ T-cells, which may contribute to the development of autoimmunity [13]. It is hypothesized that such a mechanism may have led to Hashimoto's in our patient. There is a considerable overlap in the clinical presentation of NF1 and NS, and they have often been described together as neurofibromatosis type 1/Noonan syndrome (NFNS), Watson syndrome, or LEOPARD syndrome [14]. The concomitant presentation of NF1 and NS has been documented, but their incidence remains unknown [15]. In NS, cardiac involvement is seen in approximately half of the patients, but such involvement is not seen in patients with NF1. In our patient, there were complaints of fatigue and occasional breathlessness although echocardiography did not detect any cardiac lesion expect for slightly low ejection fraction. Noonan syndrome (NS) may be a form of neurofibromatosis type 1 (NF1) since both belong to a group of clinically related disorders that share a common pathogenesis, constitutive dysregulation of the RAS-MAPK pathway with recent studies confirming a novel missense mutation in the NF1 gene in exon 24, p.L1390F, which affects the GAP-domain involved in the etiology of NFNS [16]. A growing number of cases reporting an association between neurofibromatosis type 1 and autoimmune thyroiditis point to a possible link between these etiologically different diseases. A comprehensive study using the published data from the reported cases may elucidate a veritable connection. 

## 4. Conclusion

Neurofibromatosis type 1 is a common heritable neurocutaneous disorder and is rarely associated with Hashimoto's thyroiditis. It is pertinent for a physician diagnosing neurofibromatosis type 1 to be aware of the possibility of coexisting autoimmune diseases owing to increased reports of such association. More extensive studies are required to establish whether this association with neurofibromatosis type 1 is coincidental or a link in pathogenesis does exist.
